# Alternative Treatment Positions over Supine in Adjuvant Whole Breast RT: Prone, Lateral or What Else? A Comprehensive Narrative Review

**DOI:** 10.3390/jpm16050233

**Published:** 2026-04-27

**Authors:** Ilaria Benevento, Angela Solazzo, Luciana Rago, Antonietta Montagna, Barbara D’Andrea, Fabrizio Sanna, Salvatrice Campoccia, Antonella Bianculli, Raffaele Tucciariello, Alessia Telesca, Vito Metallo, Francesco Marino, Carmen Linda Ginetti Buccino, Mario Ferrara, Irene Schirò, Teresa Virgilio, Anna Zeccola, Grazia Lazzari

**Affiliations:** 1 Radiation Oncology Unit, IRCCS CROB, 85028 Rionero in Vulture, PZ, Italy; ilaria.benevento@crob.it (I.B.); angela.solazzo@crob.it (A.S.); luciana.rago@crob.it (L.R.); antonietta.montagna@crob.it (A.M.); barbara.dandrea@crob.it (B.D.); vito.metallo@crob.it (V.M.); francesco.marino@crob.it (F.M.); carmen.ginetti@crob.it (C.L.G.B.); mario.ferrarra@crob.it (M.F.); irene.schiro@crob.it (I.S.); teresa.virgilio@crob.it (T.V.); anna.zeccola@crob.it (A.Z.); 2 Radiation Oncology Unit, San Salvatore Hospital, 07100 Sassari, SS, Italy; fabrizio.sanna@aouss.it (F.S.); salvatrice.campoccia@aouss.it (S.C.); 3 Physic Unit, IRCCS CROB, 85028 Rionero in Vulture, PZ, Italy; antonella.bianculli@crob.it (A.B.); raffaele.tucciariello@crob.it (R.T.); 4 Research Laboratory, IRCCS CROB, 85028 Rionero in Vulture, PZ, Italy; alessia.telesca@crob.it

**Keywords:** breast cancer, radiotherapy, cardiac toxicity, obesity

## Abstract

Whole breast radiotherapy in adjuvant breast cancer (BC) treatment has been commonly delivered in supine positioning for more than 50 years. Its widespread use is related to the broad availability of simple and common breast board immobilization devices, which exploit the natural recumbent position of the body with arms above the head, the good match with linac tables and well-established set up procedures. However, in scenarios like painful arm discomfort in patients with post-surgery arm or arthritic limitation, unfavorable chest geometry like pectus excavatum (PE), large and pendulous breasts in obese women and cardiac morbidity in left sided BC, supine position seems very uncomfortable for patients and troublesome for radiation oncologists. The question is how to proceed when supine IMRT or DIBH are ineffective strategies? Alternative positions have been analyzed over the past twenty years, starting with a lot of trials testing prone positioning variants with and without DIBH, lateral decubitus or upright standing radiotherapy. A better recognition of new treatment position options in BC adjuvant therapy may provide more opportunities for personalized radiotherapy in this population, to ensure they receive an appropriate, safe and comfortable treatment.

## 1. Introduction

The absolute locoregional control and survival benefit of adjuvant radiotherapy in breast cancer has been well established through multiple randomized trials, which have mostly been conducted in a supine position [1,2]. Technological advances have improved its therapeutic ratio and the quality of treatments providing more effective and safer RT delivery with breathing adaptation to reduce the cardiac and lung doses as much as possible, according to the ALARA principle [3].

Breast radiotherapy in terms of whole breast or partial breast is commonly delivered in supine decubitus. In this position, the patient is immobilized recumbent on a treatment table with arms above the head, while the beams are steered around them to arrange different treatment angles. However, not all patients are suitable for this positioning, because of unfavorable situations like post-surgery arm pain or arthritic arm impairments, pectus excavatum, left-sided breast cancer or large and pendulous breasts, as reported below. After surgery, about 60% of patients develop severe acute postoperative pain [4] that could complicate the set up in the supine position. Pectus excavatum and left-sided BC impact radiation-induced cardiac morbidities. Breast shape and size are to be taken into account for acute or late toxicities and worse cometic outcomes. Among solutions, for example, DIBH has shown to offer a better trade-off between target coverage and OAR dose-sparing, regardless of breast size [5], but not all women are the best candidates, due to obesity or cardiopulmonary comorbidities [6]. Other strategies include different partial breast techniques like Linac based APBI [7], brachytherapy [8] or IORT [9], where available. Large breasts with cup size ≥ C could be easily treated by IMRT-APBI, which has been demonstrated to offer a good reproducibility with a low rate of acute toxicity and excellent/good cosmetic long-term outcome [10]. However, several doubts have arisen in regard to clip locations and adequate margins to create a good compromise with the irradiated volume and the breast size. A higher body mass index combined with a large breast size are considered crucial factors in supine RT, because the set up on the linac table in obese women could be troublesome, and breast pendulousness is related to a high rate of acute toxicity and worse cosmetic outcomes [11]. Thus, to ensure the RT delivery in these scenarios, several alternative treatment positions to supine have been investigated in the past twenty years. Prone positioning is the most studied treatment as a valuable solution for pendulous breasts or for left-sided BC in an attempt to improve dose homogeneity in the breast or reduce the toxicity to the breast, heart, LADCA and lung and, in turn, a second cancer risk [12,13]. However, other positions have been tested like the lateral or the upright position. This review explores all these new modalities in RT delivery for BC patients, their clinical differences in toxicity and their implications for a personalized radiotherapy in the BC population, who are otherwise not compliant with supine positioning.

## 2. Material and Methods

This narrative review was performed by a comprehensive literature search of electronic databases, including PubMed, Scopus and Google Scholar. Due to the nature of the topic and the paucity of the related literature, a narrative review rather than systematic was conducted, as we needed to cover specific topics. The aim was to focus the radiation oncology community’s interest on the current knowledge and emerging modalities related to treatment position alternatives to supine in conjunction with modern technologies.

The search strategy was conducted using a combination of keywords, including “decubitus position BC radiotherapy”, “standing technique for breast radiation”, “lateral isocentring position”, “prone position versus supine position”, “upright positioning”, “large or pendulous breast radiotherapy”, and “pectus excavatum and radiotherapy”, ensuring the comprehensive identification of relevant studies. Titles and abstracts were screened for relevance, followed by a full-text review of the selected studies. Studies of interests were included if they met the following criteria: (1) published in peer-reviewed journals, (2) written in English, (3) focused on BC adjuvant radiotherapy positioning and (4) published in the last 15–20 years. Review articles, editorials, commentaries, conference papers, and response letters were omitted. Retrospective randomized studies and metanalyses referring to dosimetric reports, safety data and practical differences of alternative positioning over supine were collected and analyzed. All the previous studies and recent updates in regard to prone and lateral decubitus were included to assess their relevance with modern RT developments. Nearly 40 published studies considered eligible for this topic (25 for prone and 15 for lateral decubitus, respectively). In regard to the upright position, only 5 papers and one clinical case report were found and included, as this position is a new field of research. The arguments have been organized according to the following issues: Which conditions enable supine WB irradiation? What is the impact of unfavorable chest anatomy, breast size and shape in radiation-induced side effects on personalizing the RT positioning? What are the dosimetric and clinical advantages over supine by prone positions, lateral decubitus or upright standing? Are these solutions applicable to all RT facilities? Pros and cons are described. No AI tool was applied.

## 3. Unfavorable Factors for Supine RT Positioning

Arm impairment, unfavorable chest geometry, left laterality, breast shape and size can make the RT delivery in supine position very difficult. Severe acute postoperative pain is problematic for more than half of all breast surgery patients. It has been estimated that severe postoperative pain is symptomatic for 1 month in nearly 5% of breast surgery patients and 6–12 months in almost 10% of breast surgery patients [4]. Furthermore, obesity and older age may aggravate this effect causing a poor tolerance to the treatment position [14]. Pectus excavatum (PE) is the most common congenital chest wall deformity, recording an incidence of 1 in 300–1000 births [15]. It is an unfavorable condition in the case when WB radiotherapy is prescribed in left-sided BC. In fact, it is acknowledged that in PE, the heart and lungs may receive a significant higher radiation dose, increasing the risk of heart or pulmonary complications with 3D conformal RT because of the higher incidence of RILI related to the V30 Gy using the tangential fields [16]. Although several studies have investigated the benefit of VMAT, IMRT or Tomotherapy in this population over a 3D conformal RT in terms of lower MHD or dose to ipsilateral lung, they have failed to achieve an absolute advantage due to an increased low-dose bath to the contralateral breast and lung and in turn an increased risk of second cancers [17,18,19]. A good compromise between a Target Prioritized 3D-CRT plan (TPP) and an OAR prioritized plan (OARPP) has been found effective in obtaining a median 3 Gy MHD in these patients, but in the case of severe chest deformity, this option seems useless [20]. The risk of cardiac events related to the MHD over 4 Gy in left-sided breast cancer has been well established by Darby [21], leading to the adoption of DIBH strategies to spare the heart as much as possible, but not all patients are suitable candidate, and the DIBH systems are not available in all RT facilities. In addition, breast size and shape like large and pendulous breasts are a large concern in terms of all the RT-related toxicities (heart, lung, skin). In this regard, there are no definitive parameters to adequately define the “large” size of a breast. An empiric and practical evaluation of the USA bra size cup ≥ D for large breasts has been the most conventional feature, as adopted in the study of Pignol et al. [22]. In the past, the definition of a large breast was based on the distance between the edges of the lateral and medial fields for a 2D technique, assuming a 25 cm of breast separation for a large breast [23]. Later, with the development of modern 3D treatments, a breast clinical target volume (CTV) of ≥1.600 cm^3^ has been reallocated to the large breast size definition [24]. Furthermore, an evaluable and reproducible method to measure the breast is based on anthropometric measurements, which take into account the thickness of the left and right axillary fat and the nipple-to-pectoral muscle distance. This method has been found to correlate better with the risk of skin toxicity related to breast irradiation [25]. It is well acknowledged that the size of the breast affects the final cosmetic outcome of radiation therapy treatment and could be an important limitation in the delivery of adjuvant RT safely, as recognized by several investigators in many series including groups of large-breasted women treated in different institutions from the 1990s to date. Studies by Harris et al. [26], Clarke et al. [27], Ray et al. [28], and Gray et al. [29] have reported worse overall cosmetic outcomes with fibrosis and breast retraction in many large-breasted women treated with supine radiation. In other studies, features like edema, hyperpigmentation, teleangectasia and desquamation have been the main recorded side effects for a poor radiation-induced cosmesis outcome [30]. Thus, whole breast irradiation in supine as the standard position seems very challenging in these patients. Indeed, in the supine position, the large breast tends to fall laterally and or superiorly causing a bolus effect on the skin due to the hot spot deposition in the inframammary folds or deep axilla, where there is typically skin-on-skin contact. Given the widespread adoption of hypofractionated regimes, the role of hot spots in increasing skin toxicity should be accounted for because of the well-known phenomenon of the double-trouble and triple-trouble effects, which could amplify the delivered effective dose [31,32]. Moreover, to ensure an adequate coverage of the posterior part of the breast volume, the breast-cupped lung and heart in supine position may receive increased RT doses leading to late toxicity in terms of cardiac events and secondary malignancy risk [33]. Thus, alternative solutions could be found in the treatment positioning modifications. Over the supine decubitus, three alternative positions could be proposed: prone, isocentring lateral decubitus (ILD) and upright standing or seated position. In this review, we provide readers with effective insights into these alternative treatment positionings compared to supine, assessing their usefulness and convenience to offer a personalized whole breast irradiation.

## 4. Prone Positioning

**Figure 1:**
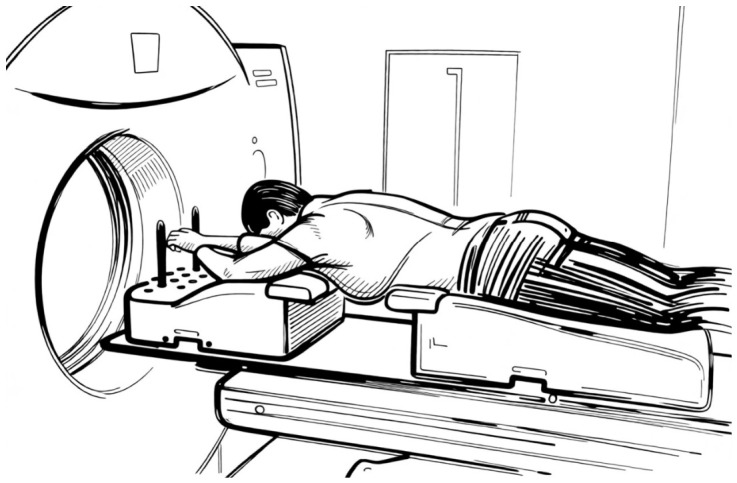
The dive prone position. Patient lies prone with arms above the head, often holding handles for stability, and the head turned to the side. The breast hangs down through an opening to isolate it from the chest wall.

**Figure 2:**
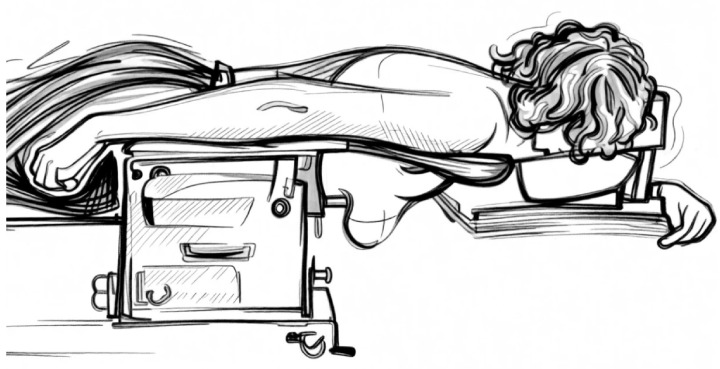
The crawl prone position from the treatment side: the patient lies prone with the treated-side arm down alongside the body and the opposite arm above the head, resembling a swimming crawl.

**Figure 3:**
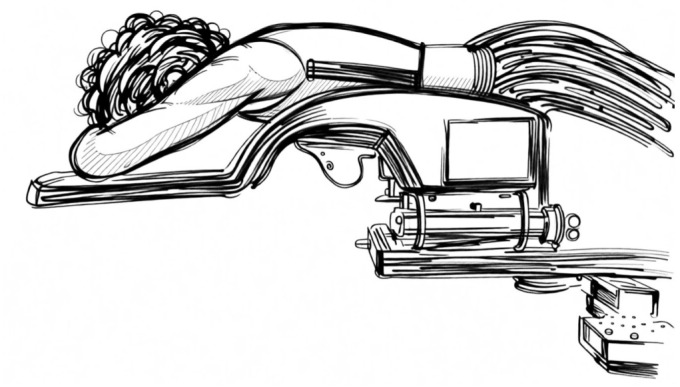
The crawl prone position from the opposite side.

Prone positioning is the most tested option and still yields a growing widespread interest. The first expectation of this position has been to improve the dose inhomogeneity in pendulous and large breasts; in turn, it has also been tested in left-sided breast irradiation to spare the heart and LADCA. In this position, the irradiated breast hangs down through an opening in the plate of a customized immobilization device to be included in the tangential RT beams. Initial experiences were carried out in a prone dive position [34,35,36] as shown in Figure 1. Later a new prone position, the so called “prone crawl”, was tested, as shown in Figure 2 and Figure 3. This position results with the arm at the treated side alongside the body and at the opposite side above the head in a swimming movement. It has been shown to be more comfortable and effective in including the nodal areas in the radiation fields [37]. Although the purpose of improving the dose homogeneity distribution in the breast has been reached, conflicting results have been achieved in regard to the advantage to the heart, LADCA and ipsilateral lung (ILL), as summarized in a meta-analysis [38]. Many trials have been conducted to improve the heart dosimetry, comparing the prone position in shallow-free breathing and DIBH RT assessing the set-up reproducibility with repeated breath hold [39]. At the beginning, only the breast was irradiated; later, the inclusion of the nodal areas was attempted in dive and crawl modalities yielding good dosimetric reports. In the dive prone position, the study of Purswuani et al. has shown the feasibility to irradiate the nodal areas after lumpectomy or mastectomy using a hybrid 3D-IMRT technique and moderate hypofractionated radiotherapy. The target volume coverages like the breast V40.5 Gy and the nodal V38.5 Gy as goals were reached in almost all cases [40]. The constraints to the OARs were also respected, and the nodal contouring delineation was guided by internal guidelines [41]. Concerning the crawl position, the studies of Speleers et al. conducted on a series of left-sided BC patients with invasive carcinoma of the breast and positive lymph node requiring nodal RT, have confirmed the feasibility to include the breast and all the axillary nodal levels and internal mammary chain. As a result, the coverage of the breast and nodal targets were similar in the supine and prone crawl positions, while the mean doses to the OARs were lower for the prone crawl than for the supine position [42]. Validated guidelines taking into account the different position of axillary nodal levels in this position have been applied and integrated into an atlas-based segmentation for crawl prone breast cancer radiation therapy [43,44].

## 5. Lateral Decubitus Positioning or Isocentring Lateral Decubitus (ILD)

**Figure 4:**
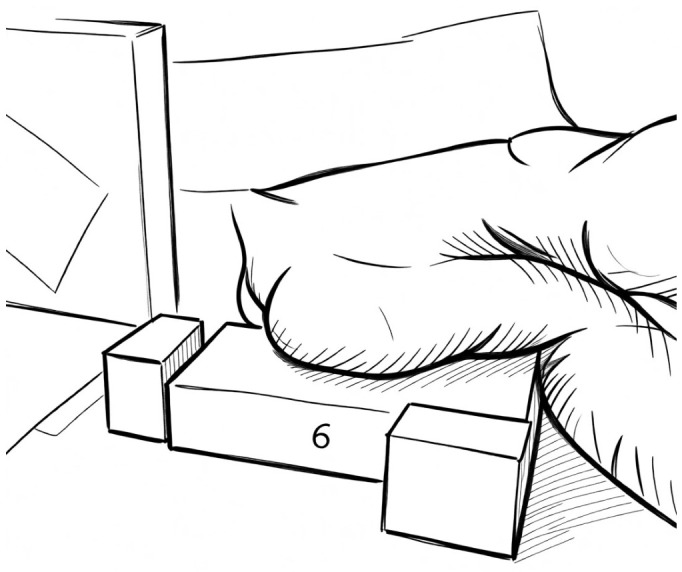
The isocentring lateral decubitus from the breast-treated side: the patient lies on the contralateral side (affected breast up) on a specialized thin carbon fiber table, ensuring the breast flattens to provide a more homogeneous thickness.

**Figure 5:**
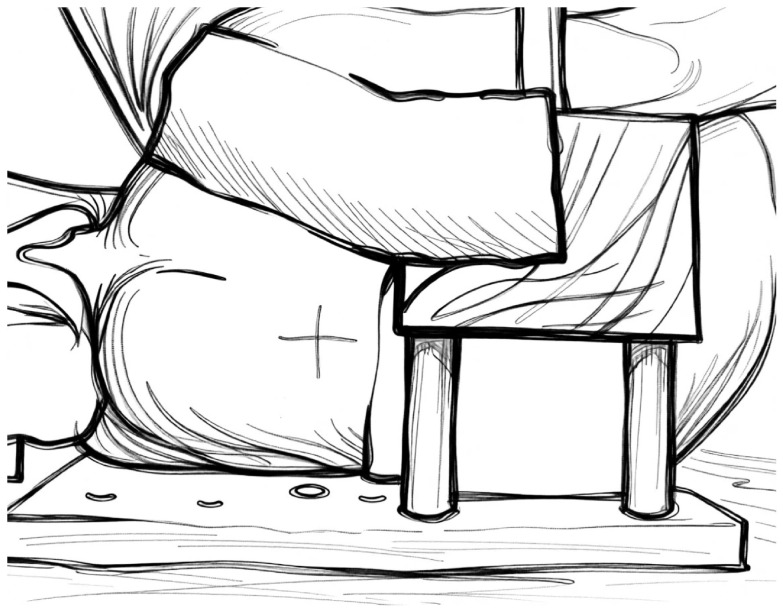
The isocentring lateral decubitus from the back with the backrest supporting the dorsal side. + is the intersection of lasers on the skin.

The breast irradiation in the lateral decubitus (LD) is a technique that was introduced at the Institute Curie more than 30 years ago with the primary end point to improve the dose homogeneity in large sized and pendulous breasts [45]. This technique was also found to be advantageous for pectus excavatum [46]. The first pivotal trial was carried out by Frangois Baclesse in the 1950s [47]; then, this modality was adopted until the 1980s on the first 644 patients treated with a 2D RT [48]. Thereafter, this treatment position was improved with a 3D isocentring technique, called “isocentring lateral decubitus (ILD)”, consisting of 3D planning and portal images to assure the correct set-up [49]. In this position, only the whole breast gland is irradiated, while the nodal areas are excluded. Usually, the patient lies with the trunk rotated from one side to the other in order to allow a treatment with an isocentring technique using two opposed photon beams tangent to the ribs wall and vertical to the breast. Initially RT was delivered with Co 60 units at an SSD of 80 cm; later, low megavoltage energies were adopted. In the historical experiences, the lateral field was delivered to the patient lying on the contralateral side to the treated breast; the ipsilateral arm had to be extended upward above the head in order to expose the breast volume to the beam. The treated breast was stretched over a support made of 3 cm lead built to protect the contralateral breast. The medial field was delivered to the patient lying on the ipsilateral side, with the ipsilateral arm below the head and the contralateral hand reclining the contralateral breast outside the beam. The breast was stretched on the treatment couch to obtain a homogeneous reduction in breast thickness, which permitted a better dose distribution in the breast parenchyma [47,48]. Later, this positioning was optimized with a reproducible setup within a customized immobilization system, as depicted in Figure 4 and Figure 5. Specific guidelines have been developed for the target delineation in this position [50]. The following contouring details have been also incorporated into a practical neural network-based autocontouring algorithm to guide a proper and time-saving planning in this decubitus [51].

## 6. The Upright Standing Positions

**Figure 6:**
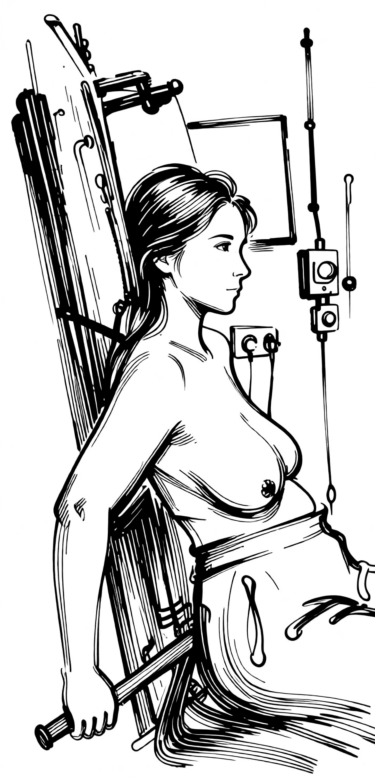
The seated upright position with arms near hips: patient is seated with arms behind the body. The individuals depicted in this figure are not actual patients; this figure serves solely as an illustrative example, and the publication of this figure does not require a patient’s informed consent.

**Figure 7:**
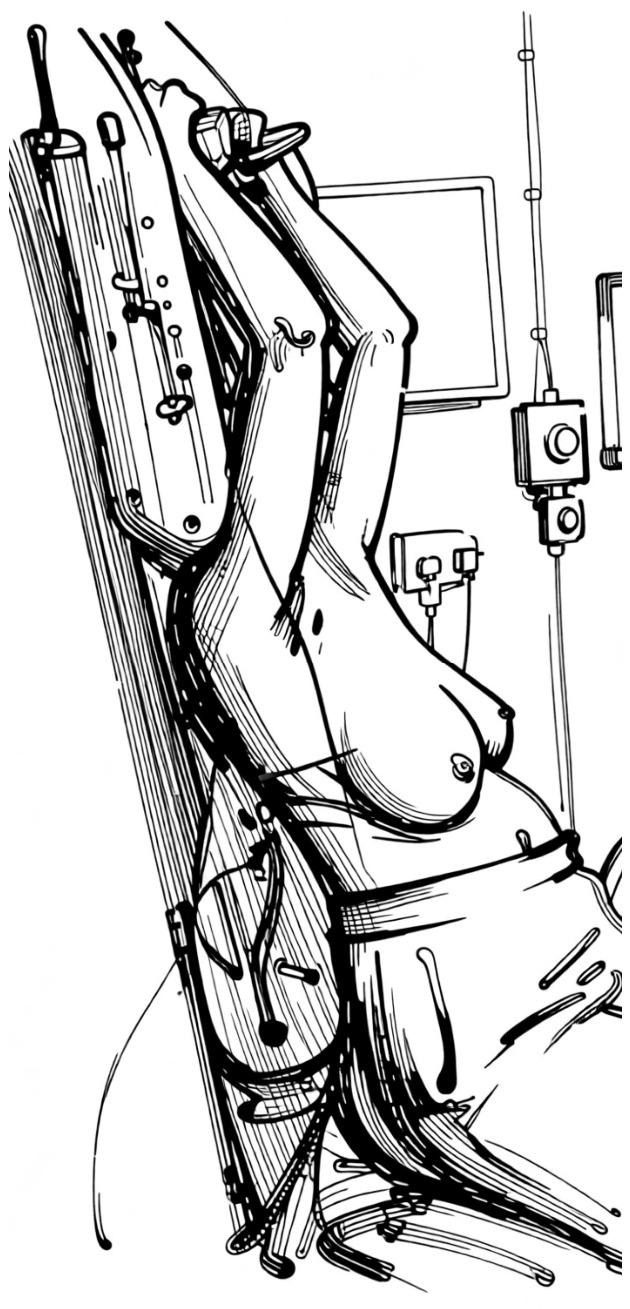
The seated upright position with arms up: patient is seated with arms up within a wing board.

This technique has been tested in obese women a with high body index exceeding the weight limitation of the equipment; recently, a novel system with rotating robotic chair with fixed gantry has been implemented using specific radiotherapy bras to smooth the inframammary fold. The first experience of upright standing RT was reported in 2010 by a team of radiation oncologists in Baltimore in USA on an obese woman with right breast cancer treated with breast conserving surgery, unable to be treated under the linac in recumbent position due to the weight limitation of the equipment [52]. The patient kept upright beyond the couch consisting of a carbon fiber SBRT body frame with the breast up on it to the level of the inframammary fold while keeping the ipsilateral arm behind her back and out of the field. As a mold, an aquaplast pelvic body cast was cut and molded over the breast. A CT scan on the empty breast mold was conducted, and planning was calculated on a tissue of equivalent density in the mold. Treatment consisted of 3D conformal RT with two opposed tangential fields and conventional fractionation. The patient was treated without disruptions with a conventional fractionated RT. This modality has a technical limitation consisting of the exclusion of the supraclavicular or axillary fields matching; thus, the irradiation of nodal areas is not permitted. Recently, this position has evolved into an upright patient positioning on a rotating robotic chair within a gantry-less linac system [53]. Since, in the seated position, the breast naturally falls vertically down, a possible anatomical advantage resulting from an upright body posture has been hypothesized, such as an increased lung volume and reduced breathing motion to exclude the use of breath holding techniques [54,55]. To smooth the inframammary breast fold and minimize the acute toxicity in the skin-to-skin contact areas, several specialized bras have been successfully adopted obtaining a safe median 3 cm reduction in the skinfold. However, this technique is still a work in progress to optimize the comfort of the patients with the setup of the trunk with the arm up or down on the flanks [53], as depicted in Figure 6 and Figure 7.

## 7. Immobilization Devices

Customized immobilization devices have been built to improve patient’s comfort, set up reproducibility, matching with modern RT delivery techniques without collisions with the equipment and or part of the patient’s body.

### 7.1. Prone Immobilization Devices

The prone dive position is the most investigated position. In this position, the head usually is resting on a forehead–chin bracket, and the arms are braced at the elbows with the hands gripping a pair of handlebars; the treated breast is hanging through an opening on the top table, while the contralateral breast is stretched on a wedged cushion, and the lower extremities are supported by a mattress [56]. Initially, the first devices consisted of a home-built wooden platform top with a hole through which the breast was hanging along the chest wall to be included within the RT field of the tangential parallel-opposed beams. Since then, several customized immobilization devices have been developed and modified overtime to make this position more comfortable and ensure a precise set up of the chest [57,58]. Another option, which has been recently developed, is the crawl prone position, so called because it mimics the crawl swimming position. In this position, the patient lies prone with the arm of the treated side alongside the body and the arm at the contralateral side above the head. A customized and very comfortable couch has been assembled to permit the irradiation not only of the breast but also of the chest wall and regional lymph nodes using a vast range of beam directions in the coronal and near-sagittal planes with IMRT or VMAT, avoiding any kind of collision with the device. This couch position has been validated in several trials by patient and physicians, and it has been defined as very comfortable and time-saving. DIBH systems are allowed for both prone modalities [59,60].

### 7.2. Isocentring Lateral Decubitus Device

As described before, in the lateral decubitus, the patient lies with the trunk rotated from one side to the other on a dedicated patient board (Techset) with a back rest. A large elastic fabric band attached to supports using Velcro serves to move the contralateral breast out of the radiation fields. The patient lies on the side of the treated breast. A thin carbon breast rest of 6 or 7 cm height with angled sides is placed under the breast with its long edge along the lateral border of the breast. The surface of this support consists of a flat part, where the breast is placed, and a curved part adapted to the convexity of the thorax. The thickness of the horizontal part is only 0.3 mm, allowing a good preservation of the skin by limiting the bolus effect. On the dorsal support, a vertical pole shaft is mounted to be used by the patient to place the contralateral arm. There is no need to add a DIBH system to spare the heart [61].

### 7.3. Upright Positioning Rotating Chair System

Upright body positioning for breast radiotherapy has not been studied extensively. The first experience was reported many years ago by Mouhiddin et al., as described before, in an obese woman not suitable for recumbent position treatment [52]. Recently, a novel study investigating a patient seated on a robotic rotating chair to deliver a seated upright breast RT modality has been conducted. In this position, the breast falls vertically down to the chest creating the inframammary fold, which needs to be smoothed out using a specific RT bra. The arm could be positioned up over the head or down on the flanks, depending on the patient’s compliance. The first trial was conducted on the ‘Eve’ linac upright patient positioner from Leo Cancer Care Ltd. ^®^ (Horley, UK). The equipment consists of a backrest, seat pan, shin rest and heel stop, which can all be adjusted independently by several angles. The initial seat pan height is set according to the patient’s lower limb length. The position of the shin rest and the heel stop are also adjusted individually, so that each patient’s lower limbs can be comfortably immobilized. This system has been tested with photons and protons, and it is still under investigation to assess how to reduce the setup errors, how to stabilize a better position of the upper body through custom-designed arm supports or which photon beam portals could be applied [62]. As reported by Yoganathan et al., in this natural position of the thorax, the organ motion for intra-thoracic organs is more pronounced in the cranio-caudal than in the antero-posterior and medial–lateral directions; thus, there is no need for breath control to spare the heart or lungs. Uncertainties in antero-posterior and medial–lateral directions influenced by respiratory motion have been measured, resulting within 3 mm in 81% and within 5 mm in 91% of the patients tested in the upright trial patients. These values seem not to impact the heart or lung dosimetry; the trial is still ongoing [63].

## 8. What Are the True Benefits in Changing the Treatment Position?

The primary end point of these alternative positions has been to reduce the dose inhomogeneity in the breast parenchyma to reduce the acute skin toxicity in the inframammary fold or in the axilla and the dose to the OARs at risk, regardless of the fractionation schedules, without compromising the target coverage and the disease control.

### 8.1. Dose Inhomogeneity and Toxicity

Dose inhomogeneity reflects a non-uniform dose distribution within the breast target volume with a significant hot spot deposition inside and outside the target volume, which is responsible for unexpected acute or late toxicity, such as occurs in large and pendulous breasts.

#### 8.1.1. Prone Positions

Many trials have been conducted to ameliorate this. A first experimental simulation on an anthropometric phantom performed by Bieri et al. showed the effect of the prone position over supine in terms of the reduction in the integral dose on OARs, with low toxicity [64]. Merchant et al., treating a group of BC patients with pendulous breasts in a prone, compared with supine position, recorded a substantial reduction in hot spots in the prone position, accounting for 102–103% versus 116–118% in the supine position, with a reduction in the dose inhomogeneity in the target by approximately 15% compared with supine tangents [56]. In a retrospective analysis of Mckinnon et al., prone positioning yielded a reduction in hot spots within 113% of the prescribed dose and the maximum average of 107.6%, recording no severe acute toxicity in the high-dose regions displacing hot spots [34]. In the study of Grann et al., a remarkable cosmetic score outcome with the prone position was recorded due to an improved homogeneity in the prone-treated large breasts, whereas in supine, the isodose distribution showed the location of hot spots at the apex of the breast and at the periphery of the breast over 20% in very large breasts [35]. Kurtman et al., when comparing the supine and prone plans in patients with large breasts, confirmed the reduction in hot spots, obtaining an improved dose homogeneity and few cases of Grade 3–4 acute and chronic toxicities [65]. In the retrospective study of Stegman et al., Grade 3 acute dermatitis and edema occurred in 2% of patients, while chronic Grade 2 to 3 skin and subcutaneous tissue toxicities were reported in 4.4% and 13.7% of patients, respectively, and a median hot spot of nearly 106% [57]. Further, in a phase-3 multicenter single-blind randomized trial on the prone vs. supine position on 378 women with large breast sizes with a bra band ≥ 40 in and/or ≥D cup, Vesprini et al. showed that the desquamation rate was higher in the group of patients treated in the supine position (*p* = 0.002) regardless of the fractionation schedules arm groups [66]. In addition, all the studies conducted by Formenti et al. on prone hypofractionated radiotherapy in APBI or WBI have provided robust data of good–excellent cosmesis using this position [36,67]. In the experiences conducted in the crawl prone on breast or chest wall and nodal areas, a lower toxicity score on the LENT-SOMA scale was recorded than that for supine positioning (*p* = 0.005) [68]. Although hypofractionation in five fractions over 10–12 days led to a significantly less retraction, telangiectasia and pain 2 years after irradiation, in the following overview of the five-year photographic assessment, no significant difference in worsened breast cosmesis was observed between the groups [68].

#### 8.1.2. The Isocentring Lateral Decubitus

By the pioneering experience described by Forquet, the first clinical result was the reduction in the breast thickness, as confirmed by CT scans conducted in the first trial. The breast thicknesses measured from skin to skin on a theoretical beam axis was 13 cm on the dorsal position and 7.5 cm on the lateral position; they were 19 and 9 cm at the deepest edge of the field, respectively [45]. This effect had a good impact in reducing the acute toxicity as observed in the following study conducted at Institute Curie, accounting for more than 500 patients treated in LD and with two opposed fields tangent to the chest wall with photons 4–6 MV at SAD 100 cm or 60 Co at SAD 80 cm [48]. In the study of Campana et al., a low toxicity rate was reported through a comparative study among the three known breast irradiation techniques using a 60 Co unit vs. the conventional dorsal decubitus technique (DD) using 4 MV photons or 60 Cobalt vs. ILD technique using 5 MV photons [49]. In the study conducted by Kyrova et al., the first observed effect was the reduction in breast separation, measuring between 8 cm and 22.5 cm, with an average of 12.25 cm to allow the use of low energy photons. The target coverage was adequate in all patients with a good dose homogeneity according to the ICRU criteria of 5% and +7%. In this study, 37.5% of patients experienced Grade 1 dermatitis, while in 58.9%, Grade 2 dermatitis was recorded, and only one patient showed Grade 3 dermatitis. The cosmetic outcome was good in 76.8% of cases, while a fair outcome was recorded in 3.5% of patients [69]. In the set of BC patients with pectus excavatum, this technique allowed the same breast dose homogeneity as in the supine technique with a decreased breast thickness of 4.5–6.8 cm. Acute skin toxicity was inconsistent for all patients at completion of ILD radiotherapy [46]. Hypofractionated schedules could influence the risk of toxicity as recorded in the study of Bronsart, where several fractionation schemes from conventional to START A-B and weekly five fraction schedules were tested. After a median time of 4 weeks, acute dermatitis was recorded in 93% of the entire cohort. Grade 3 dermatitis was observed in only 2.8% of cases. The hypofractionated schedule of 30 Gy in five fractions was significantly related to an increase in the fibrosis rate (*p* = 0.0011) compared to the standard scheme of 50 Gy/25 fr [70].

#### 8.1.3. Upright Position

No data are available, because the assessment of this position is still under investigation outside clinical trials. However, in the experience of Mouhiddin, the patient was treated without interruptions developing a grade 2 hyperpigmentation by week 4 and a Grade 2 inframammary desquamation by week 5 healed with silvadene by week 7. At 6 months follow-up, the breast was soft with normal skin [52].

### 8.2. Disease Outcomes

In regard to data on local control and survival outcomes in prone position radiotherapy, all the above studies have provided evidence of long-term disease control similar to the supine treatments in prone and lateral decubitus positions.

#### 8.2.1. Prone Positions

In the study of Stengman et al., after a median follow-up of 4.9 years, the 5-year actuarial true local and elsewhere ipsilateral breast tumor recurrence rates were 4.8% and 1.3%, respectively. The 5-year actuarial rates of regional nodal recurrence and distant metastases were 1.6% and 7.4%. Actuarial disease-free, disease-specific, and overall survival rates at 5 years were 89.4%, 97.3%, and 93%, respectively [57]. In the study of Formenti et al., no ipsilateral breast local recurrence, regional nodal recurrence, or contralateral breast cancer were observed, resulting in a 3-year breast cancer-specific survival of 95.6% [67]. Data on long term outcomes in the crawl position are a work in progress and are related to an SIB-hypofractionated RT in five fractions. However, in the longitudinal analysis of a phase 2 open-label trial including 100 patients with large breast size randomized between prone and supine positioning, the 5-year overall survival was 96% in both groups [68].

#### 8.2.2. The Isocentring Lateral Decubitus

Regarding the ILD, the overall survival and relapse-free interval have been reported by an analysis of Xua et al. on 832 consecutive patients. With a median follow-up of 6.4 years, only 36 patients experienced locoregional recurrence, and no association with the fractionation regimen was identified (*p* = 0.2). Among these relapsed patients, only 28 pts had “in-breast” local recurrences, two had local recurrences and regional lymph node recurrence, and six had regional lymph node recurrence only in non-irradiated areas. The median time to recurrence was 50 months. The complete mapping of patterns of recurrences was performed, and, in most cases, local recurrences were situated adjacent to the primary tumor bed. Multivariate Cox regression analysis showed that the site of recurrence had no significant impact on the overall survival (*p* = 0.14) [71]. In an upright seated position, no data are available.

## 9. What Is the Benefit to Organs at Risk?

Another key point requiring the adoption of alternative positions is to minimize the dose to the heart, LADCA and ipsilateral lung in left-sided BC patients, who are unable to perform DIBH, regardless of the breast size. While there is no agreement on an advantage on this topic in trials using the free breathing prone position, studies with ILD seem to have hit the target very well with a surprising unknown effect like the estimated dose reduction to circulating immune cells (EDIC), which is a new organ at risk to consider. At this moment no dosimetric data are available for the upright seated position.

### 9.1. Prone Position

Free or shallow breathing prone positions have been hypothesized to be able to reach this end point in dive and crawl positions as well over the supine position; however, no substantial differences have been recorded, and disappointing data have been shown. In fact, Griem et al. found no differences for heart V30 Gy and V20 Gy (*p* = 1.000) over supine, probably due to the anatomical downshift of the lateral and superior border of the heart close to the chest wall [72], as confirmed by Chino’s measurements. In this study, the authors calculated the distance between the heart and chest wall at nine points in three axial levels of the sternum in both supine CT and prone MRI, discovering a mean 19 mm of systematic displacement in the lateral and superior border of the heart closer to the chest wall in the prone MRI images vs. CT scan images acquired in the supine position. The mean displacement was 19 mm (*p* < 0.001) with a significant reduction in lung volume between the heart and chest wall in the prone series (*p* < 0.001), thus resulting in no benefit on the heart dose [73]. On the contrary, the study of Gregucci et al. reported several dosimetric advantages for the heart in terms of the mean values for MHD = 0.69 Gy, LADCA Dmean = 2.20 Gy, and LADCA Dmax = 4.44 Gy 41, in a comparison with data provided by the current literature on hypofractionated radiotherapy and EQD2 conversion [74]. In the trials conducted in crawl prone positioning on equal terms of target volume coverages with the supine decubitus, the dose to OARs were significantly lower (*p* < 0.05) for the ipsilateral lung, contralateral lung, thyroid, esophagus and skin, while again, no significant differences in heart doses were reached [59]. The sparing dose effect of DIBH in the prone position has been evaluated in the metanalyses of Lai et al. [38]. In this metanalysis, the data of 751 patients by 19 observational studies investigating the dose–effect relationship on OARs between supine (S-FB) versus dive prone in free breathing (P-FB) and DIBH were collected and analyzed. As a result, compared with the S-FB, the P-FB allowed a significant benefit in the dose reduction to the heart, left anterior descending coronary artery (LADCA), and ipsilateral lung (ILL) (*p <* 0.00001), while no significant difference in target coverage between the S-FB and P-FB groups (*p* = 0.66) was found [38]. Among these studies, the study of Mulliez et al. showed a significant advantage to the heart in S-DIBH (*p* < 0.001), while in the P-FB this benefit was significant to LADCA and ILL (*p* < 0.001) [75]. On the contrary, in the study by Saini et al., the P-FB differences in heart and LADCA dosimetry between the two positions were not confirmed, although a dose reduction to ILL was significant (*p* < 0.001) [76]. However, in the comparison of P-DIBH and S-DIBH, in the meta-analysis, both studies agreed on the dosimetric advantage in the heart, LADCA, and ILL (*p* < 0.001), for P-DIBH over S-DIBH [38]. In a following confirmation study on prone SB vs. DIBH, in this last technique, significantly, the mean dose to the heart as in photon and proton plans was decreased. The mean heart dose reduction in DIBH (compared with SB) for photon and proton was on average 2.0 Gy (range: −1.0–3.5) and 0.56 Gy RBE (range: 0.1–1.1), respectively. The left lung mean dose decreased by approximately 13%. Meanwhile, in proton DIBH plans, the average mean left lung dose increased by approximately 21%. No significant difference was observed for proton or photon in the mean dose to the contralateral breast [77].

### 9.2. Isocentring Lateral Decubitus

All data collected on patients treated in ILD at the Curie Institute are in agreement with the reduction of dose to OARs at risk, as shown in the paper of Campana et al., who reported a reduction dose of 10% of the prescribed dose to the lung and heart due to the beam fitted nearly tangent to the chest wall. The authors concluded that this technique could have been the only solution to irradiate large and pendulous breasts while IMRT was still under development [49]. Similarly, Kyrova et al. confirmed this advantage consisting of an average mean ipsilateral 0.96 Gy lung dose and an average 17.6 Gy maximal dose. Moreover, parameters V2 Gy and V5 Gy resulted 9.3% and 0.7%, respectively. In patients with left-sided tumors, very low heart doses were recorded with an average mean heart dose of 1.34 Gy, while doses to the contralateral breast and other organs were not relevant [69]. In the study conducted in the series of patients with pectus excavatum, the differences for the lung and heart were substantial; in fact, in the supine positioning, the width of the lung and/or heart receiving >20 Gy ranged between 2.1 cm and 4.3 cm; on the contrary in ILD, this parameter ranged between 0.5 cm and 1.1 cm. Moreover, while the estimated percentage of ipsilateral lung receiving >20 Gy ranged from 21% to 34% in the supine technique, this value ranged from 0% to 5% in ILD [46]. This dosimetric advantage seems to involve not only the whole heart but also various cardiac substructures. In fact, through a recent comparative study, the mean dose to the heart, to the various cardiac structures (left ventricle, left coronary, right coronary), to the homolateral lung, and to the contralateral lung have been significantly lower in the isocentring lateral decubitus than in dorsal decubitus. As a result, the average absolute mean dose reductions have been −40 cGy for the heart, −27.5 cGy for the left ventricle, −56.5 cGy for the right coronary artery, −64.5 cGy for the left coronary artery, −45.5 cGy for the sinoatrial node, −74 cGy for the homolateral lung, and −4.5 cGy for the contralateral lung [78]. Another novelty to take into consideration is the impact of ILD on the estimated dose to circulating immune cells (EDIC). In fact, it seems that, with this positioning, it is possible to induce a potential immune-sparing effect on EDIC by modifying the beam orientation very close to the chest wall in order to reduce the incidental exposure of immune-related structures like the heart, lungs, and liver, which contribute to the estimated dose to circulating immune cells (EDIC) [79]. In a retrospective study conducted by Cheptea et al., in ILD, the calculated EDIC value was significantly lower than in the supine position (median 0.56 Gy vs. 1.12 Gy; *p* < 0.01), with a significantly lower dose to the lungs, heart and liver [79].

## 10. Discussion

The supine position is the most common modality applied to deliver adjuvant breast radiotherapy in BC patients worldwide. However, not all patients are suitable candidates for this modality because of clinical or anatomical factors, which make it troublesome. The aim of this comprehensive narrative review is to focus on alternative validated solutions in uncomfortable scenarios, in which the supine position is difficult to adopt, in order to assure a comfortable RT delivery, improve dose homogeneity to the target and minimize the dose to OARs. Efforts like the use of higher energy photons, a field-in-field intensity modulation [80], and breath-hold protocols have been tested as effective solutions for the majority of BC patients [3]; however, not all BC patients are eligible for these modern techniques. In addition to arm pain, which is a very limiting factor in maintaining a position with the arm above the head, mainly for elderly patients, overweight and obesity are also factors putting patients at a higher risk of a long-term radiation-induced pain [81]. Large or pendulous breasts are a large challenge because of severe acute skin reactions in the axilla or inframammary fold, due to a bolus effect or increased lung dose, particularly in cases where the breast extends laterally beyond the midaxillary line [82]. Acute skin side effects in large and pendulous breasts and the following worse cosmetic outcome are expected features, due to inhomogeneous dose distribution; thus, the toxicity could be amplified by hot spots leading to a double- and triple-trouble effect with a hypofractionated radiotherapy schedule [83], which is the standard fractionation [84]. A large breast size and heart toxicity in left-sided BC patients are closely connected factors; in fact, in left BC, a large breast size has been found to be related to an increased mean and maximum point heart dose [85,86] as well as the breast’s shape [87]. Moreover, the total dose of radiation, young age and the irradiated volume of normal tissue may increase the risk for secondary lung, esophageal, or thyroid cancer, and secondary sarcomas [88,89,90]. Thus, the dose optimization concept to minimize the dose, as declared by the acronym ALARA, should be pursued, considering that, compared to the general population, BC patients have an increased risk of secondary non-breast cancers five or more years after BC diagnosis with and without radiation therapy (RR 1.12; 95% confidence interval 1.06–1.19) [90]. A novel strategy to overcome these criticisms and deliver adjuvant RT safely could be the positioning modification over the supine, as depicted in this review. The prone positioning and lateral decubitus are both excellent strategies in improving the homogeneity dose distribution into the breast, reducing the breast thickness and avoiding hot spot depositions in the folds with a consequent lower skin toxicity [66,69]. The inframammary fold is completely smoothed in the upright position by using the specific RT bra; moreover, in the upright position, it is possible to assure a more comfortable arm position on the flank or above the head while the patient is seated [62]. The crawl prone position offers better arm comfort and allows nodal irradiation with several beam arrangements in IMRT, whereas this item is not possible in ILD. In regard to the dose at OARs, all these positions seem effective in lowering the dose in different ways: in the prone position, the heart could be spared with DIBH; in ILD, the beam arrangements close to the chest are angled to avoid the heart with a consequent advantage on EDIC. The upright position offers a low organ motion in the cranio-caudal direction, as occurs with the normal breath [63]; thus, it exploits the anatomical position of the heart far from the beam, and probably, a DIBH technique could be avoided. At this moment, no data are available. Overall, these techniques, each for its own characteristics, seem to be effective options but require well-trained staff and expensive equipment according to the financial policy of each RT utility. A summary of the specific characteristics is provided in Table 1.

## 11. Conclusions

Although supine decubitus remains the standard position in WB radiotherapy, alternative solutions to treat patients in particular scenarios not suitable for this modality have been found effective and safe. In fact, post-surgery arm pain, obesity, large and pendulous breast, and cardiac morbidity may affect the RT supine delivery. A prone position and ILD are the most adopted strategies, because they have been found to be very comfortable and helpful; however, these techniques require well-trained staff and expensive equipment. The seated upright solution, which could solve the arm impairment, needs more sophisticated equipment like a robotic seated gantry free Linac, bras to reduce the breast skin fold and a validated protocol to solve several criticisms like the exclusion of the contralateral breast from the treatment fields and arm immobilization away from the fields, with customized arm supports and a stable set up of the trunk. Taking into account the advantages and the specific requirements in each position and in the absence of randomized trials assessing which one is better, adjuvant WBI in critical scenarios when the supine position is not suitable can be is assured by one of these personalized treatment positions.

## Figures and Tables

**Table 1:** Summary of advantages and technical characteristics related to topics.

Topic	Prone	Isolateral	Upright
Large and pendulous breast, pectus excavatum, arm discomfort	Allowed; crawl: better arm comfort	Allowed	Allowed; RT bra is necessary
RT techniques	2D; 3D conformal; IMRT-VMAT (crawl) with or without DIBH	2D-3D isocentring; no DIBH	On study with protons; no DIBH
Target	Breast, chest wall nodes in crawl	Only breast	Only breast
Immobilization devices	Dive and crawl coaches	Techset coach	SBRT table coach; rotating seat gantry free linac and RT bras
Dose inhomogeneity	Reduced hot spots; G2-G3 toxicity low	Reduced breast thickness Reduced breast separation distance; G2-G3 toxicity low	Inframammary fold smoothing
Disease control	5 yrs LRR = 4.8% 5 yrs OS = 93–96%	At 6 yrs, LRR 4.3% (breast and nodal)	No data
Organ-at-risk benefits	Heart, no differences; advantages in heart, LADCA, ILL, cl-breast; P-DIBH better	10% reduction dose to heart, lung, cl-breast EDIC-reduced dose	No data

## Data Availability

No new data were created or analyzed in this study. Data sharing is not applicable to this article.

## Abbreviations

ALARA	As Low As Reasonably Achievable.
APBI	Accelerated Partial Breast Irradiation
BC	Breast Cancer
cL-Breast	Contralateral Breast
Co 60	Cobaltum 60 Unit
CT	Computed Tomography
CTV	Clinical Target Volume
2D	Two Dimensional
3D	Three Dimensional
DIBH	Deep Inspiration Breath Hold
EQD2	Equivalent Dose in 2 Gy
ICRU	International Commission on Radiation Units and Measurements
ILL	Ipsilateral Lung
IMRT	Intensity-Modulated Radiation Therapy
IORT	Intraoperative Radiation Therapy
LADCA	Left Anterior Descending Coronary Artery
LENT-SOMA scale	Late Effects on Normal Tissue—Subjective, Objective Management and Analytic scale
Linac	Linear Accelerator
MHD	Mean Heart Dose
MRI	Magnetic Resonance Imaging
MV	Megavolt
OAR’s	Organs at Risk
PE	Pectus Excavatum
RILI	Radiation-Induced Lung Injury
RT	Radiotherapy
SAD	Source Axis Distance
SBRT	Stereotactic Body Radiation Therapy
SSD	Source Skin Distance
V × Gy	Volume Receiving xGy
VMAT	Volumetric Modulated Arc Therapy
WBI	Whole Breast Irradiation
